# A comparison of the metabolic effects of treadmill and wheel running exercise in mouse model

**DOI:** 10.1186/s42826-019-0035-8

**Published:** 2020-02-07

**Authors:** Youn Ju Kim, Hye Jin Kim, Won Jun Lee, Je Kyung Seong

**Affiliations:** 1 Laboratory of Developmental Biology and Genomics, BK21 Program for Veterinary Science, College of Veterinary Medicine, Seoul National University, Seoul, South Korea; 2 The Research Institute for Veterinary Science, College of Veterinary Medicine, Seoul National University, Seoul, 08826 Republic of Korea; 3 Korea Mouse Phenotyping Center (KMPC), Seoul National University, 08826 Seoul, Republic of Korea; 4 Severance Biomedical Science Institute, Yonsei University College of Medicine, Seoul, 03722 South Korea; 5 Interdisciplinary Program for Bioinformatics, Program for Cancer Biology, BIO-MAX/N-Bio Institute, Seoul National University, 08826 Seoul, Republic of Korea

**Keywords:** Exercise, Treadmill, Wheel running, Physiology, Adipocyte, Muscle fiber

## Abstract

Aerobic exercise is well known to have a positive impact on body composition, muscle strength, and oxidative capacity. In animal model, both treadmill and wheel running exercise modalities have become more popular, in order to study physiological adaptation associated with aerobic exercise. However, few studies have compared physiological adaptations in response to either treadmill exercise (TE), or voluntary wheel running exercise (WE). We therefore compared each exercise intervention on body composition and oxidative markers in male C57BL/6 N mice. The total distance run was remarkably higher in the WE group than in the TE group. Both forms of exercise resulted in the reduction of body weight, fat mass, and adipocyte size. However, the average for grip strength of WE was higher than for control and TE. Interestingly, PGC-1α expression was increased in the gastrocnemius (glycolytic-oxidative) and soleus (oxidative) muscle of TE group, whereas WE showed a significant effect on PGC-1α expression only in the soleus muscle. However, muscle fiber type composition was not shifted remarkably in either type of exercise. These results suggest that TE and WE may exert beneficial effects in suppressing metabolic risks in mouse model through attenuating body weight, fat mass, size, and increase in mitochondria biogenesis marker, PGC-1α.

## Introduction

It is well known that regular exercise can have a substantial positive effect on various health conditions [1]. In particular, aerobic exercise has emerged as an effective prevention and treatment for metabolic problems [2]. Therefore, many researchers have tried to utilize the treadmill exercise (TE) or wheel running exercise (WE) in mouse and rat model to detect various physiological and metabolic responses [3–8]. As is commonly known, TE is required exercise at the appointed time and intensity, while WE is voluntary enhanced activity in mice. However, it is not clear which form of exercise training is more appropriate for the challenges in the study of metabolic changes by aerobic exercise. Our study aimed to compare the effect of 8-weeks of TE and WE training on the basic physiological and metabolic parameters, such as body composition, grip strength, skeletal muscle mitochondrial biogenesis marker (PGC-1α), and skeletal muscle fiber type in male C57BL/6 N mice model.

## Materials and methods

### Animal and experimental design

The 7 weeks-old Male C57BL/6 N mice were purchased from Central Lab. Animal Inc. (Seoul, Korea). Mice were randomly divided into the following groups: control (CON, *n* = 5), treadmill exercise (TE, n = 5), and wheel running exercise (WE, n = 5). Mice were maintained at temperature of (22–24) °C, humidity of (50–60) %, with a 12 h light/dark cycle in a specific pathogen-free barrier facility, and had ad libitum access to a regular chow diet (NIH-31, Ziegler Bros, PA), along with tap water. All animal experimental protocol was performed according to the “Guide for Animal Experiments” (Edited by the Korean Academy of Medical Sciences) and approved by the Institutional Animal Care and Use Committee (IACUC) of Seoul National University (Approval Number SNU-160718-3-4).

### Treadmill and wheel running exercise protocol

Before the exercise training, 1 week of adaptation was followed for the TE group mice to become familiarized to the treadmill (Columbus Instruments, Ohio). After the adaption period, a 5 days/week progressive exercise training regime was utilized, such that the speed and intensity were incrementally increased from 60 min at 17 m/min in week 1 to 60 min at 24 m/min by week 8 of training, with the incline of the machine being gradually raised from (5 to 15°) during exercise periods. The WE group performed voluntary wheel running exercise for the same periods, for 8 weeks. The distance of voluntary running per day was recorded by activity wheel running machine. (Activity wheel, TECNIPLAST, Italy).

### Grip strength

The grip strength of all mice was measured for maximal muscle strength. Mouse grasped a steel greed connected to a force gauge. Then the mice’s tail was pulled against the steel greed, until its forelimb and hind limb released the steel greed. The force (g) was measured three times, and the maximum grip strength value was used for analysis. Grip strength was measured using a Grip Strength Meter (Bioseb, Vitrolles Cedex, France) at the last week (week 8) of the experiment.

### Body composition

Fat and lean body masses were assessed by ^1^H magnetic resonance spectroscopy after TE and WE. Body Composition was analyzed by Nuclear Magnetic Resonance (NMR) methods (Minispec LF-50, Bruker BioSpin, MA).

### Western blotting

Total proteins were extracted using PRO-PREP buffer (iNtRON Biotechnology Inc., Seoul, Korea) containing proteinase inhibitors and phosphatase inhibitors (GenDEPOT, Barker, TX). Homogenates were centrifuged at 13,000 rpm for 15 min at 4 °C, supernatant were collected, and protein concentration was determined using the BCA protein assay kit (Thermo Scientific, Rockford, IL). Equal amounts of protein were resolved on SDS-PAGE gels, and then transferred to PVDF membranes. Primary antibodies against the following proteins were used: PGC1α (Abcam, Cambridge, UK), Troponin I-SS (C-19), Troponin I-FS (G-7) (Santa Cruz Biotechnology, CA, USA), and GAPDH (Cell Signaling Technology, MA, USA). The membranes were then incubated with anti-rabbit or anti-mouse IgG horse-radish peroxidase-linked secondary antibody (AbClon, Korea), and then visualized with Micro-Chemi 4.2 system (DNR Bio Imaging Systems, Israel). The target protein levels were then normalized against the GAPDH protein levels. Band intensities were measured with image J software (NIH, USA).

### H&E staining

Tissues were weighed, and fixed with 4% paraformaldehyde (Biosesang, Korea) at room temperature (RT) overnight. Paraffin-embedded sections of fat were sliced at thickness of 3 μm. Paraffin sections of fat tissues were deparaffinized, and stained with Hematoxylin & Eosin (H&E), following standard procedures. Sectioned tissues were analyzed under a scanner (Pannoramic Scan, 3D HISTECH) and Image-Pro program.

### Statistical analysis

All values were performed using Prism 7 software. Data were expressed as the mean ± SEM. Statistical analysis was performed using One-way ANOVA between groups. Turkey’s post hoc test was performed to express the mean difference between groups. *p* < 0.05 was considered statically significant.

## Results

### Comparison of running characteristics of the treadmill and wheel running exercise

Table 1 shows that animals exercised significantly longer on WE than on TE. The total distance increased gradually in the (2nd – 5th) weeks of training in TE mice, reaching a plateau by weeks 6–8. In WE mice, the running distance increased rapidly in the 2nd week of training, and decreased gradually until weeks 4–8.

**Table 1:** Weekly running distances in C57BL/6 N mice for 8 weeks after initiation of TE and WE

	TE		WE
Weeks	Distance (km)	Incline (°)	Distance
1	3.90	0	166.10 ± 29.40
2	4.40	0	232.42 ± 43.51
3	5.02	0	216.71 ± 44.02
4	5.87	0	160.18 ± 29.82
5	6.25	5	146.80 ± 39.69
6	6.41	10	154.06 ± 31.55
7	6.42	15	155.47 ± 39.72
8	6.44	20	126.32 ± 41.18

### The effect of treadmill and wheel running exercise on body weight, body composition, fat weight, and food intake

**Fig. 1:**
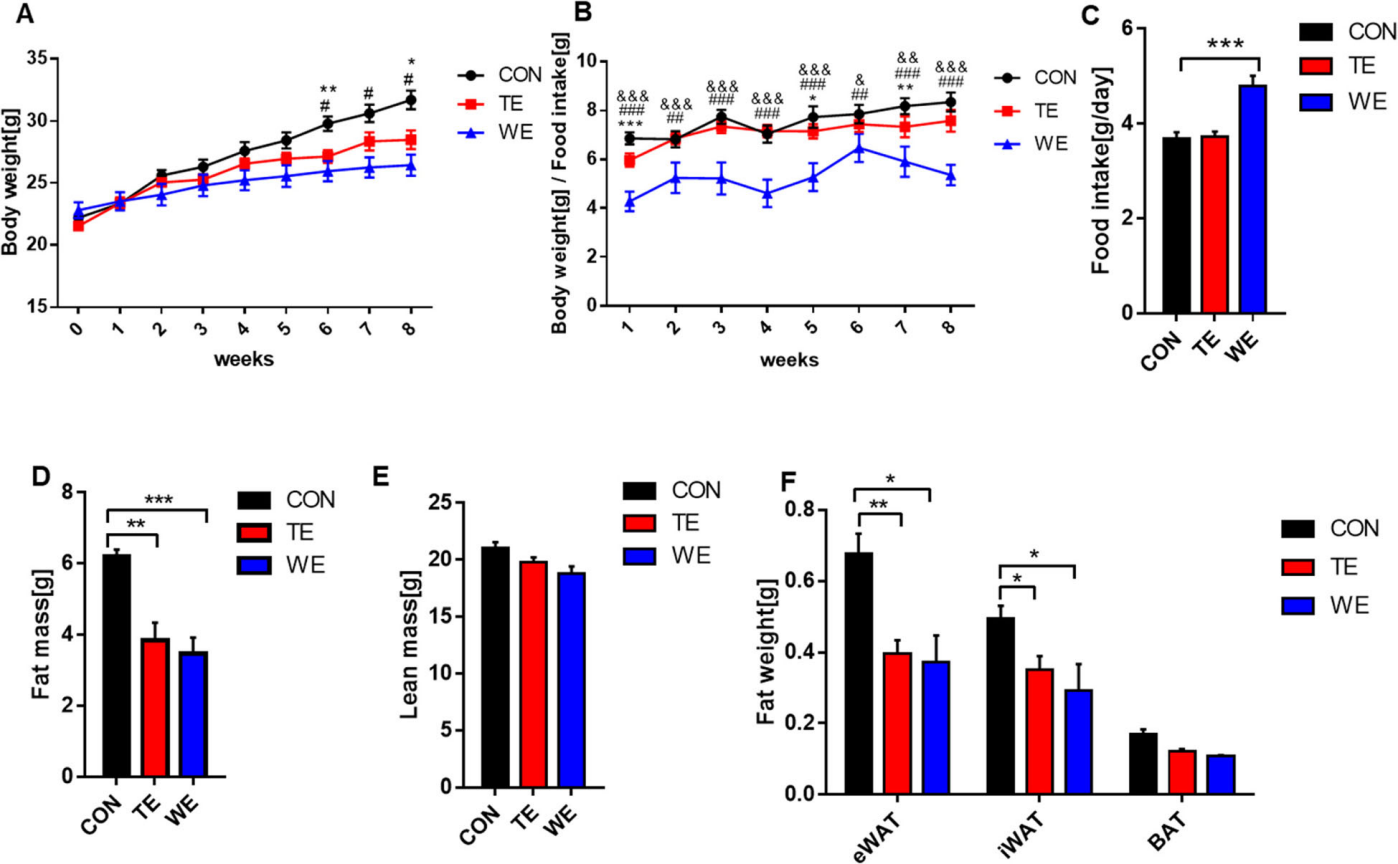
Effect of treadmill running and voluntary wheel running on body weight, food intake, body composition, and fat weight. **a** Body weight gain, **b** Body weight / food intake per week, **c** Food intake per day, **d** and **e** Body composition analysis by NMR spectroscopy, and **f** Fat (eWAT, iWAT, BAT) weight. Data are presented as the mean ± SEM; *n* = 5 per group. Significance level set as *p < 0.05; **p < 0.01; ***p < 0.001. CON, Control; TE, Treadmill Exercise; WE, Wheel Running Exercise; *Compared CON vs TE; ^#^Compared to CON vs WE, ^&^Compared to TE vs WE

Significantly decreased body weight (*p* < .05) was recorded in both the TE and WE groups after 8 week of treatment, compared to those in the CON group (Fig. 1a). Interestingly, food Intake per day of the WE group was the highest compared with those of TE and CON groups, although WE mice had the lowest body weight (Figs. 1b and c). Nuclear Magnetic Resonance (NMR) recorded significantly decreased fat mass in both the TE (*p* < 0.01) and WE (*p* < 0.001) animals after 8 weeks of training, compared to those in the CON animals (Fig. 1d). However, lean mass was not changed by TE and WE (Fig. 1e).

Consistent with this result, eWAT and iWAT weights were significantly lower in both TE (eWAT; *p* < 0.01, iWAT; *p* < 0.05) and WE (eWAT and iWAT; *p* < 0.05) groups, compared to that in the CON group. However, BAT weight was not significantly lower in the TE and WE groups, compared to that in the CON group (Fig. 1f).

### Effect of treadmill and wheel running exercise on skeletal muscle weight and grip strength

**Fig. 2:**
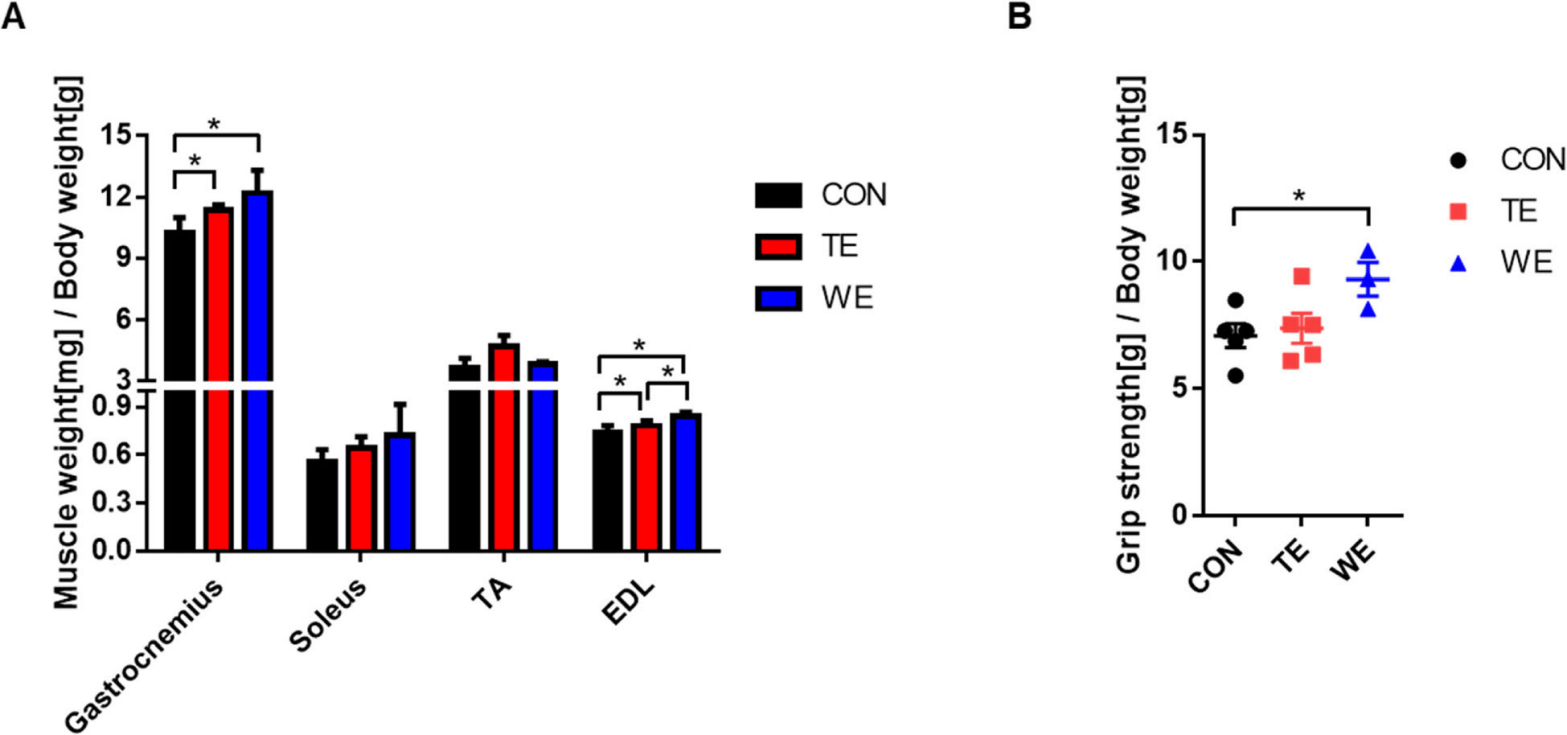
Effect of treadmill running and wheel running on skeletal muscle weight and grip strength. **a** Skeletal muscle (gastrocnemius, soleus, TA, and EDL) weight, and **b** Grip strength. Data are presented as the mean ± SEM; *n* = 5 per group. Significance level set as *p < 0.05. *Significantly different from the following lines. CON, Control; TE, Treadmill Exercise; WE, Wheel Running Exercise

Figure 2a shows that significantly increased muscle weight / body weight was recorded in the TE (Gastrocnemius and EDL; *p* < 0.05) and WE (Gastrocnemius and EDL; *p* < 0.05) groups, compared to those in the CON group. In addition, significantly increased EDL muscle weight / body weight was recorded in the WE group, compared to that in the TE group. Next, we determined whether increase in muscle weight was associated with increased muscle strength. The grip strength analysis revealed that grip strength per body weight was increased significantly in the WE group, compared to in the CON group. However, it was not considerably increased in the TE group, compared with that in the CON group (Fig. 2b).

### Treadmill and wheel running reduces adipocyte size

**Fig. 3:**
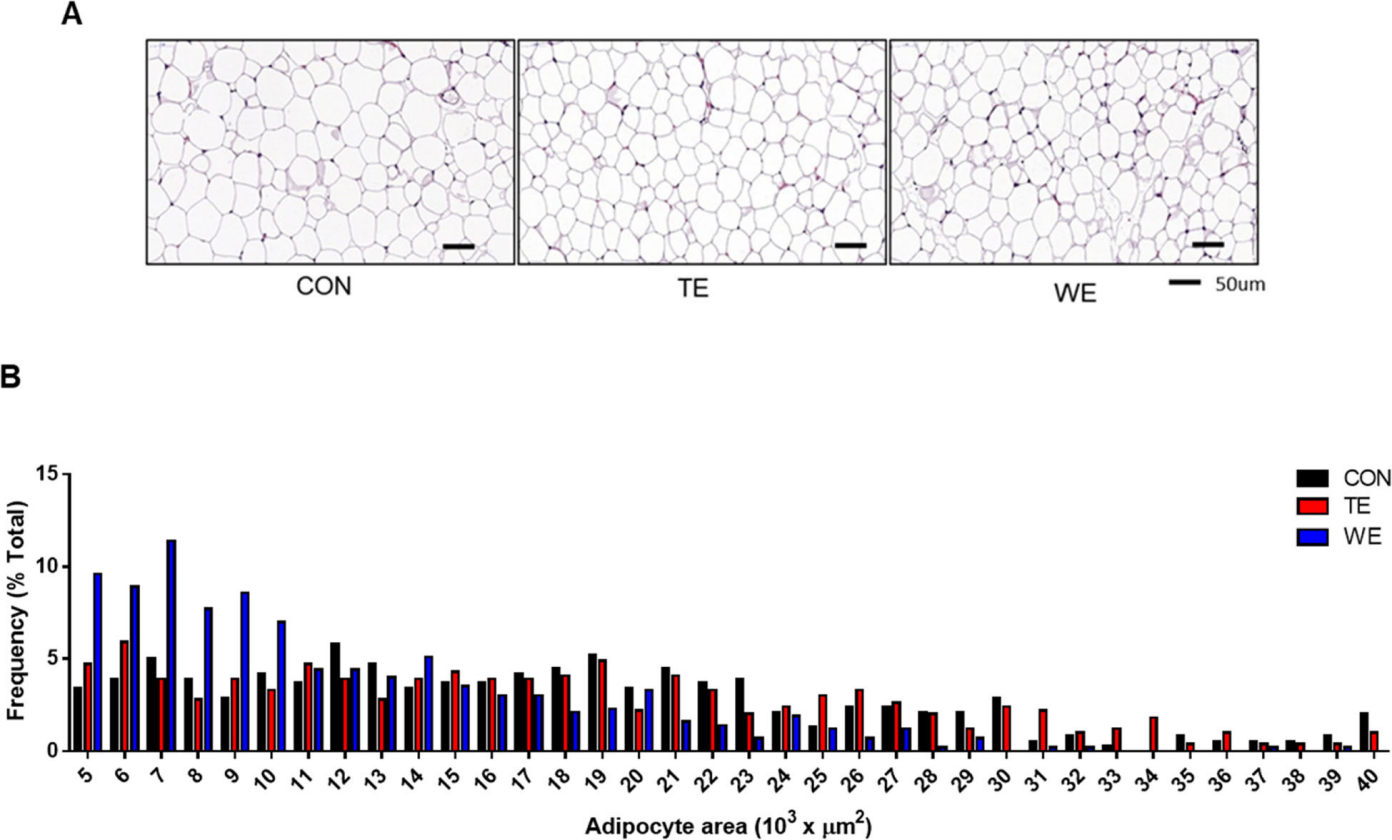
Epididymal white adipose tissue (eWAT) section analysis after 8 weeks of treadmill and wheel running. **a** Representative images of eWAT sections stained with H&E (scale bar size is 50 μm), and **b** Adipocyte size distribution Frequency (%) counted by Image-Pro. CON, Control; TE, Treadmill Exercise; WE, Wheel Running Exercise

Histological analyses also revealed that adipocyte (eWAT) size were decreased in both TE and WE groups (Fig. 3a). In addition, the frequency (%) of adipocyte distribution was lower among TE and WE groups compared to CON group (Fig. 3b). However, they were reduced remarkably in the WE group, compared to that in the TE group.

### Effect of treadmill and wheel running exercise on mitochondria biogenesis

**Fig. 4:**
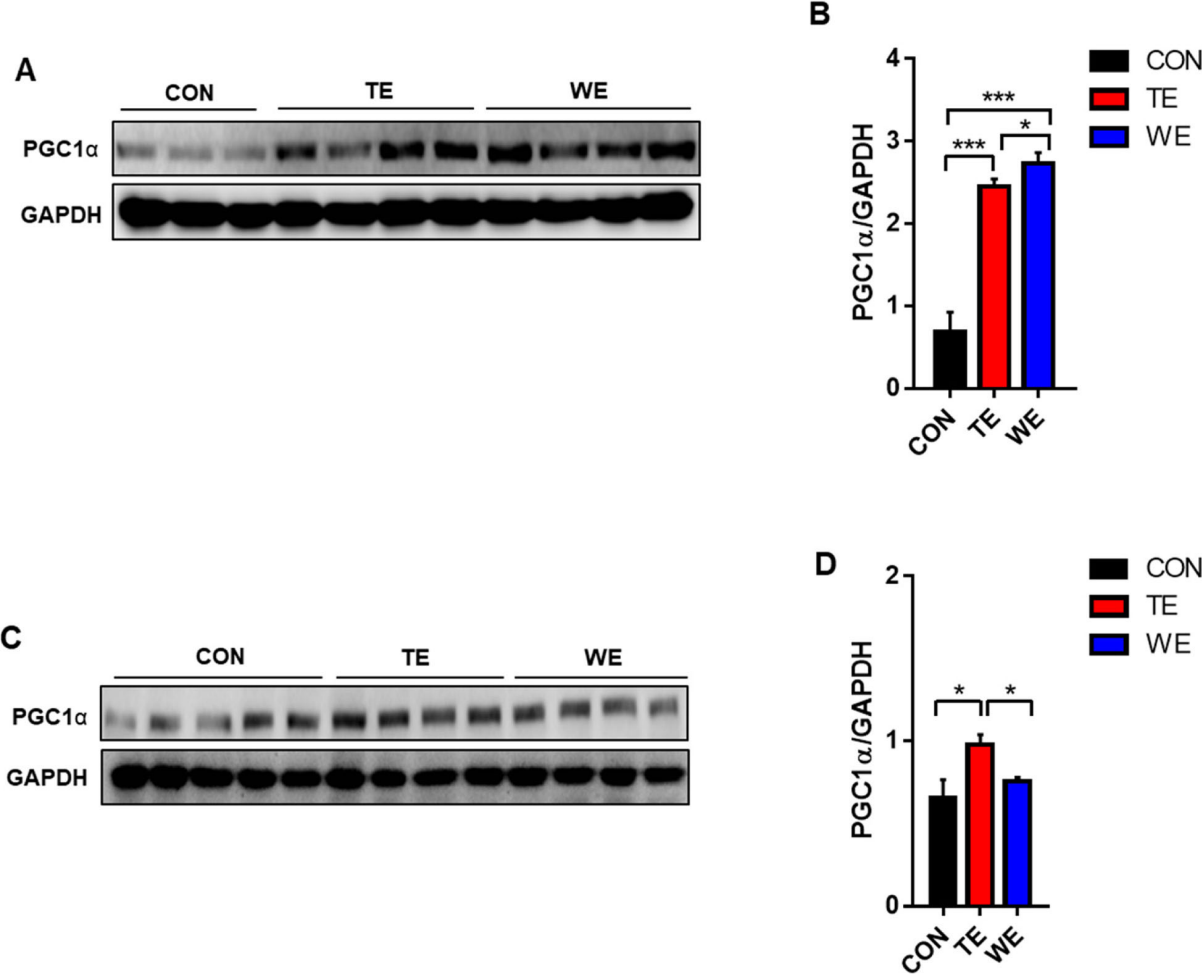
Expression of mitochondrial biogenesis marker, PGC-1α in skeletal muscle. Expression of PGC-1α in soleus muscles (**a**) and (**b**). Expression of PGC-1α in gastrocnemius muscles (**c**) and (**d**). Data are presented as the mean ± SEM; *n* = (3–5) per group. Significance level set as *p < 0.05; ***p < 0.001. *Significantly different from the following lines. CON, Control; TE, Treadmill Exercise; WE, Wheel Running Exercise

To further investigate the process involved in fat mass reduction, peroxisome proliferator-activated receptor γ coactivator-1α (PGC1α) protein expression in the soleus and gastrocnemius muscle were determined. PGC1α protein expression in the soleus (oxidative) muscle was significantly increased by TE and WE (both; *p* < 0.001), compared to that in the CON group (Fig. 4a and b). However, PGC-1α protein expression in the gastrocnemius (glycolytic-oxidative) muscle showed increase only in the TE group, compared to in the CON group (*p* < .05) (Fig. 4c and d).

### Effect of treadmill and wheel running exercise on skeletal muscle fiber type shifting

**Fig. 5:**
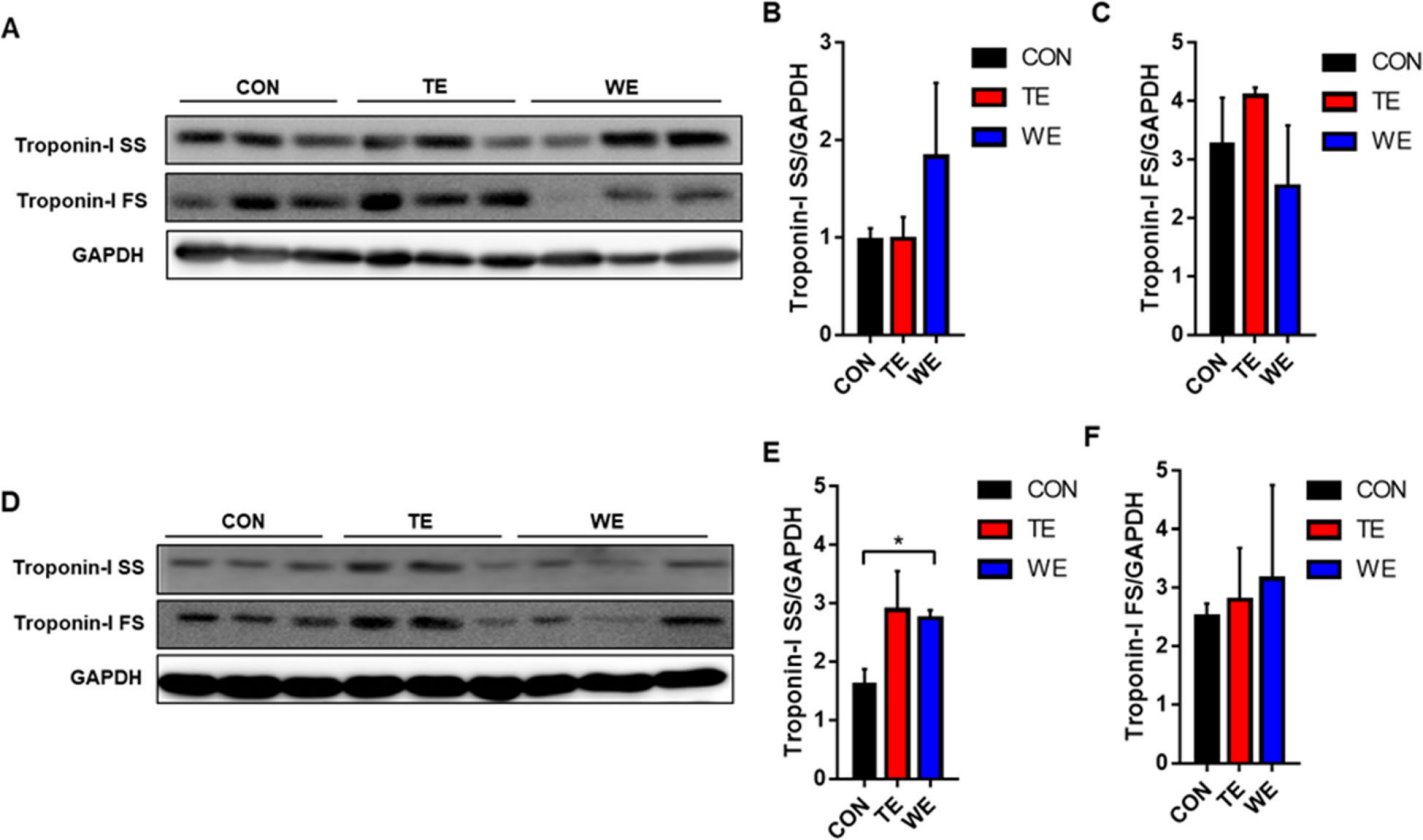
Effect of Treadmill and Wheel Running Exercise on fiber type changes in skeletal muscles. Troponin 1-SS (Slow skeletal muscle twitch fibers, Type1 fiber) and Troponin 1-FS (Fast skeletal muscle twitch fibers, Type2b fiber) expression levels in soleus muscles (**a**), (**b**) and (**c**), gastrocnemius muscles (**d**), (**e**), and (**f**). Data are presented as the mean ± SEM; *n* = 3 per group. CON, Control; TE, Treadmill Exercise; WE, Wheel Running Exercise

The effect of TE and WE training on fiber type shift was then investigated using antibodies specific to the Troponin I isoforms Troponin I-FS (type2, white muscle), and Troponin I-SS (type1, red muscle), which are common marker proteins of distinct muscle fiber types. Troponin 1-SS is usually marked in slow-twitch oxidative fiber, such as soleus muscle. In contrast, Troponin I-FS is usually marked in fast-twitch glycolytic fiber, such as EDL. In our study, we determined whether increase in Troponin I-SS was associated with increased exercise-induced oxidative capacity. This analysis revealed that the expressions of Troponin I-SS and Troponin I-FS proteins were not significantly changed in both soleus and gastrocnemius muscle (Fig. 5a-d).

## Discussion

The present study compared the impact of either TE or WE on body composition, muscle strength, muscle size, fat size, and oxidative capacity of skeletal muscle in C57BL/6 N mice. This study yielded several main findings.

First, in terms of reducing body weight and fat size, both TE and WE are effective exercise modalities. This effect was the largest in the WE group, although the food intake of the WE group was the highest among groups. These results might be due to the fact that the exercise volume of the WE group was much higher than that of TE. In terms of distance, WE mice ran roughly (20–40) times longer. Although the TE group ran much less than the WE group, the magnitude of changes in body composition after TE was similar to those observed in WE. It is known that voluntary wheel running, unlike forced treadmill running allows the animal to freely exercise with minimal or no external stress [9]. Involuntary treadmill exercise is known to stimulate release of cortisol [10]. Acute elevation of cortisol after physical exercise stimulates metabolism and catabolism. Therefore, increased level of cortisol induced by stressful involuntary treadmill exercise might be the reason for TE group to have a similar extent of decrease in weight and fat mass observed in the WE group.

Many studies have demonstrated that in response to increased energy demand, exercise-trained athletes and animals increase food intake [11, 12]. Furthermore, Koteja et al. (1999) found that food consumption per body mass was positively associated with the number of revolutions run per day [13]. Based on the fact that our results confirmed that the WE mice consumed more food per day than the CON and TE mice, we also investigated whether a chronic aerobic exercise training increase in weight loss would promote loss of skeletal muscle mass, because loss of grip strength is strongly associated with loss of body weight, muscle mass, and strength [14]. To answer this question, we performed measurement of muscle mass/body weight and grip strength. Interestingly, grip strength was significantly elevated in the WE group. These results might be due to the fact that although the absolute value of grip strength was similar between groups, the relative value of the grip strength of the WE group was significantly higher than that of the other groups, because of the lowest body weight of the WE group.

Second, both TE and WE had no effect on muscle fiber type composition in the soleus and gastrocnemius muscle. Aerobic exercise training adaptation is characterized by changes in skeletal muscle contractile and structure protein expression toward a more oxidative fiber composition that is better suited for metabolic improvement [15, 16]. However, in the current study, both types of exercise training could not alter the muscle fiber type composition of the glycolytic-fast and oxidative-slow muscle.

Third, muscle oxidative capacity determined by PGC-1 α was significantly affected by both TE and WE in oxidative muscle. It is well known that PGC-1α is a key regulator of skeletal muscle mitochondrial number and function, as well as an increase in oxidative muscle fiber [17]. In addition, PGC-1α has been suggested to be an important factor in mediating exercise training-induced adaptations in mitochondrial biogenesis [18]. Our results suggest that increased fat oxidation through the induction of PGC-1α by both TE and WE might be partially responsible for the significant reduction of fat size and mass in the TE and WE groups. The remarkable phenomenon was the elevation of PGC-1α expression in gastrocnemius (glycolytic-oxidative) muscle of TE mice, but not in WE mice. This result suggests that the intensity of WE was much lower in comparison to that of TE, in order to recruit type II muscle fiber. In fact, although gastrocnemius muscle is classified as type II muscle, it is actually composed of a mixture of oxidative and glycolytic fiber. Therefore, it is possible that the WE performed in our study might not be sufficient to induce mitochondrial biogenesis through the PGC-1a in glycolytic-oxidative muscle fibers.

It has been known that physiological changes induced by voluntary wheel running were often qualitatively similar, but may often be quantitatively less robust than those achieved by forced treadmill exercise, which is typically done at higher speed and inclination. However, the results of the current study show that physiological adaptations from both TE and WE were similar in terms of reducing body composition and fat size, and increasing muscle mitochondrial biogenesis, because mice undergoing voluntary WE ran substantially farther per night than TE groups. Therefore, although the intensity of forced TE was much higher than that of WE, the greater overall volume of exercise by WE appeared to be sufficient to produce similar adaptational responses.

## Conclusion

Our results revealed that both TE and WE contribute to the maintenance of metabolic health. However, the total distance of wheel running exercise is relatively high when compared to forced treadmill exercise. Thus, it is important to consider the different factors that can affect the activity and outcomes of both TE and WE.

## Data Availability

The data that support the findings of this study are available on request from the corresponding author on reasonable request.

## Abbreviations

BAT: Brown Adipose Tissue
CON: Control
EDL: Extensor Digitorum Longus
eWAT: Epididymal white adipose tissue
FS: Fast skeletal muscle twitch fibers
iWAT: Inguinal White Adipose Tissue
PGC-1 α: Peroxisome proliferator-activated receptor γ coactivator-1α
SS: Slow skeletal muscle twitch fibers
TA: Tibialis Anterior
TE: Treadmill Exercise
WE: Voluntary Wheel Running Exercise
